# Comprehensive Analysis of TRIM Family Genes in Hepatitis Virus B-Related Hepatoma Carcinoma

**DOI:** 10.3389/fgene.2022.913743

**Published:** 2022-07-07

**Authors:** Wei Hu, Dongsheng Liu, Renjie Li, Hong Qian, Wei Qiu, Qingwang Ye, Fanyun Kong

**Affiliations:** ^1^ NanJing Drum Tower Hospital Group Suqian Hospital, The Affiliated Suqian Hospital of Xuzhou Medical University, Suqian, China; ^2^ Department of Pathogenic Biology and Immunology, Laboratory of Infection and Immunity, Xuzhou Medical University, Xuzhou, China

**Keywords:** hepatitis B virus, hepatocellular carcinoma, prognosis, immune infiltrates, bioinformatics analysis, TRIM

## Abstract

**Background:** As significant components of E3 ligases, the tripartite motif (TRIM) proteins participate in various biological processes and facilitate the development of several diseases. Nevertheless, the correlations of TIRMs with hepatitis B virus (HBV)-positive hepatoma carcinoma (HCC) are not well elaborated.

**Methods:** The expression profile of TRIM genes in HBV-associated HCC and related clinical information were extracted from the Cancer Genome Atla (TCGA) database and the International Cancer Genome Consortium (ICGC) database. Dependent on the ConsensusPathDB and STRING databases, the gene ontology, Reactome pathways, and protein-protein interaction were assessed. Relied on TIMER 2.0 database, the relationship of the TRIMs with immune infiltration was investigated. Using multivariate analysis and Kaplan Meier analysis, the association between TRIM genes and the prognostic value was examined.

**Results:** A total of 17 TRIM genes, including TRIM16, TRIM17, and TRIM31 with fold change no less than 1.5, were discovered to upregulate in HBV-associated HCC in both TCGA and ICGC cohorts. Relied on gene enrichment analysis, the identified TRIMs were observed to not only be related to the interferon and cytokine signaling but also linked to the adaptive immune system. Particularly, the co-expression patterns of identified TRIMs with other E3 ligase genes and many innate immune genes that are associated with Toll-like receptor signaling, apoptosis, and SUMOylation. Besides, some of identified TRIM expressions were also linked to the infiltration levels of T cells and B cells. Additionally, several TRIM genes were associated with various clinical factors and relevant to the poor survival of HBV-associated HCC.

**Conclusion:** Our findings could deepen our understanding of TRIMs and their correlations with HBV-associated HCC. Furthermore, some of these TRIMs may be utilized as new prognostic markers of HBV-related HCC prognosis, or act as potential molecular targets for the disease.

## Introduction

In the eastern Pacific region and Africa, the chronic infection of hepatitis B virus (HBV) is still a predominant risk factor for the initiation and development of hepatoma carcinoma (HCC) (Geng et al., 2015; Jia et al., 2020; Arslan et al., 2021; Jiang Y. et al., 2021). The occurrence and progress of HCC have a close association with various cellular factors regulated by HBV. Particularly, increasing evidence has implicated that HBV X protein (HBX) and its C-terminal truncated mutations are capable of modulating gene transcription and protein degradation of host factors to control several biological processes, including cellular proliferation, autophagy, migration, apoptosis, and metastasis to facilitate hepatocarcinogenesis (Ali et al., 2014; Kim et al., 2016). Furthermore, the integration of viral DNA into the human chromosome is capable of causing gene instability and change the expression of numerous vital host genes, including oncogenes and non-coding RNA genes, to accelerate HCC progression (Bousali et al., 2021; Zhang et al., 2021). Additionally, the interaction of HBV with the host immune system could induce chronic inflammatory conditions to favor the generation of an immunosuppressive microenvironment to induce tumorigenesis (Trehanpati and Vyas, 2017; Timperi and Barnaba, 2020; Cho and Cheong, 2021). Considering the significance of cellular factors in initiating and promoting tumorigenesis induced by the virus, further exploring novel factors related to HBV-related HCC could help us develop new strategies to treat the disease.

The tripartite motif (TRIM) proteins, which contain a highly conserved really interesting new gene domain, the B-Boxes domain, as well as the coiled-coil domain, are vital constituents of E3 ubiquitin ligases (Zhao et al., 2021). To date, TRIM has been identified to be a large human protein family, and no less than 80 TRIM proteins have been discovered. Especially, the TRIM proteins have been identified to participate in lots of biological processes, such as antiviral infection, gene transcription, differentiation, signal transduction, proliferation, DNA repair, and immune response (Jin and Zhu, 2021; Mohammadi et al., 2021; Roy and Singh, 2021). Furthermore, the altered expression of TRIM proteins contributes to the development of several diseases, such as cardiovascular diseases, infectious diseases, autoimmune diseases, and different malignant cancers (Zhang et al., 2020; D'Amico et al., 2021; Roy and Singh, 2021; Zhao et al., 2021). Recently, the reported researches have indicated that TRIM proteins also contribute to hepatocarcinogenesis (Zhang et al., 2018; Fan et al., 2019; Liu et al., 2020). Besides these, lots of TRIMs also have the capability of modulating the replication of HBV (Gao et al., 2009; Zhang et al., 2013; Mu et al., 2020). However, until now, a comprehensive understanding of the relationship of TRIMs with the development of HBV-associated HCC is still not well investigated.

In the present study, relied on TCGA and ICGC cohorts, we comprehensively estimated the expression pattern of TRIMs in HBV-associated HCC, assessed the biological functions and pathways related to TRIMs, and further evaluated the association of these TRIMs with other E3 ligases, innate immune genes, the infiltration levels of immune cells, clinical-pathological features, as well as the prognosis of patients with HBV-related HCC. Our results could help deepen the comprehension of the underlying mechanisms related to TRIMs in tumorigenesis induced by the virus.

## Materials and Methods

### Data Collection

The information on the expression patterns of TRIM genes from HBV-associated HCC was obtained from The Cancer Genome Atlas (TCGA) (TCGA-LIHC cohort, HBV-related HCC, *n* = 84; adjacent tissues, *n* = 50) database and the International Cancer Genome Consortium (ICGC) (ICGC-LIRI-JP cohort, HBV-related HCC, *n* = 53; adjacent tissues, *n* = 45) database. The data relating to sequence-based mRNA expression and somatic mutation of HBV-associated HCC, and associated clinical information were available in these two cohorts. The mRNA levels from TCGA and ICGC cohorts were gotten from RNA sequencing, based on Illumina Hiseq platforms. Additionally, the mRNA levels of TRIMs in the TCGA cohort regarding to HBV-associated HCC tissues and adjacent tissues were acquired from the Genomic Data Commons (GDC) website, based on Sangerbox tools (http://sangerbox.com). The mRNA levels of TRIMs in the ICGC cohort were retrieved in HCCDB database (Lian et al., 2018). The somatic mutation data related to HBV-associated HCC in the TCGA cohort, which was obtained by whole-exome sequencing, was downloaded from the cBioPortal database (Gao et al., 2013). Besides these, the somatic mutation information of HBV-associated HCC was acquired by Illumina HiSeq or Illumina GA sequencing platform and retrieved from the ICGC database (Zhang et al., 2019). The present study was approved by the ethics committee of NanJing Drum Tower Hospital Group SuQian Hospital.

### The Enrichment Analysis Related to Gene Ontology and Pathway

GO analysis was applied to estimate the molecular functions of identified TRIM genes. Reactome pathway was performed to estimate the significant biological pathways related to the enriched TRIM genes. Moreover, GO, as well as Reactome pathway analyses were applied, dependent on the ConsensusPathDB database (http://cpdb.molgen.mpg.de/). Additionally, for the enrichment analysis associated with GO and Reactome pathway, the minimum enriched TRIM genes were ≥ 2, and *p* < 0.05 was significant.

### Protein-Protein Interaction Network Construction and Mutation Driver Gene Analysis

The PPI information of identified TRIMs was gotten from the STRING database (Franceschini et al., 2013), and relied on Cytoscape v3.2.1 software (Shannon et al., 2003), the interaction networks were visualized. The analysis related to the mutation driver gene was performed with the OncoVar database (Wang et al., 2021). Besides these, the co-expression analysis of identified genes was assessed by Sangerbox tools, |R|>0.05, as well as *p* < 0.05, were examined significantly.

### Identification of Innate Immune Genes, E3 Ligase Genes, and Immune Cells Analysis, and the Validation of TRIM Genes

The innate immune genes were extracted from the InnateDB database (Breuer et al., 2013). E3 ligase genes were identified in an online database (https://esbl.nhlbi.nih.gov/Databases/KSBP2/Targets/Lists/E3-ligases/) created by Medvar, et al. The estimation of immune cell infiltration in HBV-associated HCC tissues in both TCGA and ICGC cohorts was used with the TIMER 2.0 database (Li et al., 2020). To validate the expressions of identified TRIM genes, the human gene expression cohorts, including GSE77509, GSE94660, GSE55092, and GSE121248, which were gotten from the Gene Expression Omnibus (GEO) database, were used (Barrett and Edgar, 2006). GSE77509 with 18 normal samples and 18 HBV-related HCC samples, and GSE94660, consisting of 21 normal samples and 21 HBV-associated HCC samples, were acquired from RNA-sequencing relied on Illumina Hiseq 2500 platforms. GSE55092 contains 91 non-tumor tissues and 49 HBV-associated HCC tissues. A total of 37 adjacent normal samples and 70 HBV-associated HCC samples were in GSE121248. The gene expression information of these two cohorts was examined by Affymetrix Human Genome U133 Plus 2.0 Array. To verify the mutation of identified TRIM genes, the somatic mutation information was acquired from the LICA-CN cohort, which contains 402 patients with HBV-related HCC in the ICGC database.

### Statistical Analysis

An independent sample t-test was applied to assess the expression levels of different TRIMs between HBV-associated HCC and adjacent tissues, with Excel and SPSS 19.0 software. Depending on the Heml software (Version 1.0), the results were visualized with the heat map. To determine the effect of different TRIMs on overall survival (OS), as well as disease-free survival (DFS), HBV-positive HCC cases were divided into high or low TRIM expression groups, depending on the median value of identified TRIM levels. Additionally, the survival analysis was applied with the Cox regression model in multivariate analysis, or with the Genhan-Breslow-Wilcoxon test in Kaplan Meier analysis. Pearson coefficient was utilized for the correlation between immune cells or immune checkpoints and different TRIMs. The differences were significant when the *p-*value < 0.05.

## Results

### The Expression Characteristics of TRIM Genes in HBV-Associated Hepatocellular Carcinoma

**FIGURE 1:**
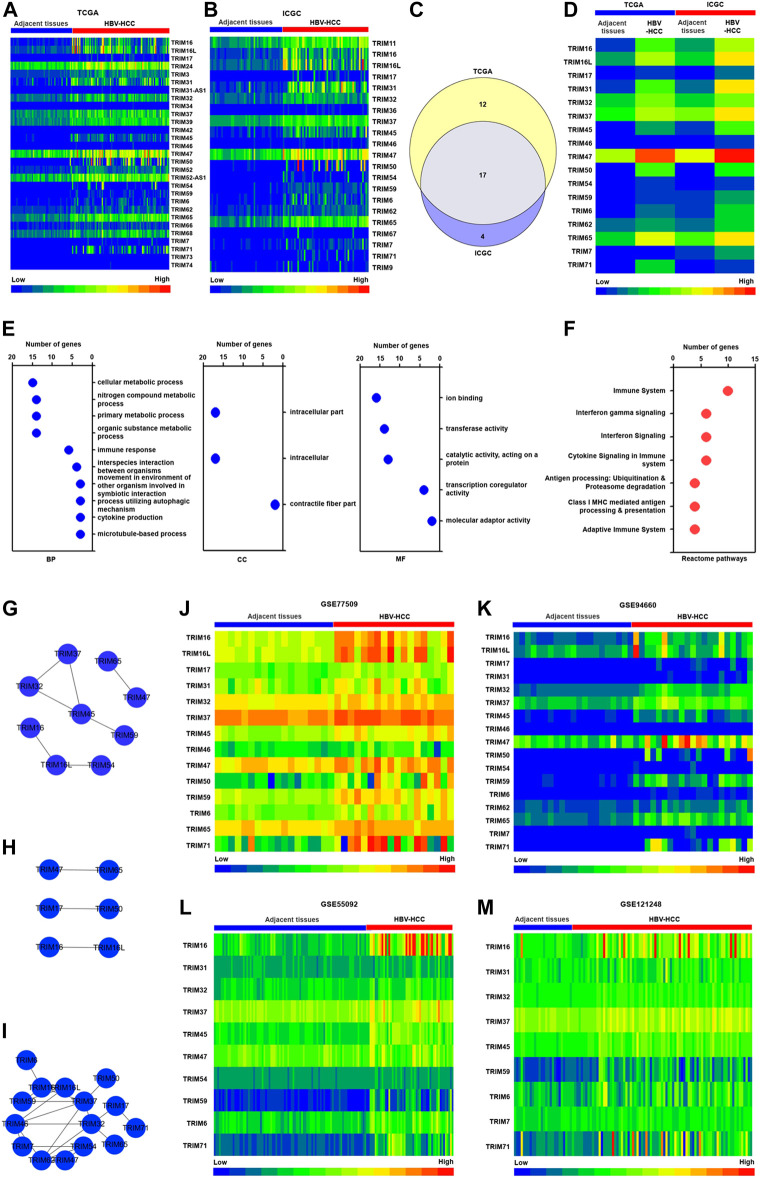
The characteristics of TRIM gene expression in HBV-positive HCC. **(A)** The upregulated TRIM genes in HBV-associated HCC in the TCGA cohort. **(B)** The upregulated TRIMs in HBV-positive HCC in the ICGC cohort. **(C)** The common TRIM genes in HBV-positive HCC in TCGA and ICGC cohorts. **(D)** The expression of common TRIM genes in HBV-positive HCC in both two cohorts. **(E)** The GO analysis of common TRIM genes. **(F)** The Reactome analysis of common TRIM genes. **(G)** The co-expression pattern of identified TRIMs in HBV-positive HCC in the TCGA cohort. **(H)** The co-expression pattern of identified TRIMs in HBV-positive HCC in ICGC cohort. **(I)** The interaction network of identified TRIMs in HBV-positive HCC. **(J)** The expression of identified TRIMs in the GSE77509 cohort. **(K)** The levels of identified TRIMs in the GSE94660 cohort. **(L)** The expression of identified TRIMs in the GSE55092 cohort. **(M)** The expression pattern of identified TRIMs in the GSE121248 cohort.

Firstly, we assessed the expression profiles of TRIM genes in the TCGA and ICGC cohorts, in which the gene expression levels were gotten from the RNA sequencing, based on Illumina Hiseq platforms. The results showed that compared to adjacent tissues, 29 TRIM genes were discovered to be significantly upregulated in HBV-associated HCC tissues in the TCGA cohort (The fold change was no less than 1.5 and *p* < 0.05) (Figure 1A). A total of 21 upregulated TRIM genes with fold change ≥ 1.5 were discovered in the HBV-positive HCC tissues in the ICGC cohort, compared with adjacent tissues (*p* < 0.05) (Figure 1B). We also analyzed the common TRIM genes in HBV-related HCC between these two cohorts. As revealed in Figures 1C,D, 17 common upregulated TRIM genes were found in TCGA and ICGC cohorts. Next, these common TRIM genes were selected for further investigation. To determine the biological functions link to identified common TRIM genes, Gene enrichment analysis with GO was conducted, and 18 GO terms were discovered (Supplementary Table S1). Dependent on the number of TRIMs, the top 10 enriched GO terms were presented. The results of the GO analysis indicated that the identified TRIMs were associated with various biological processes (BP), molecular functions (MF), as well as cellular components (CC). As the results displayed in Figure 1E, the enriched GO terms of BP were linked to nitrogen compound metabolic process, cellular metabolic process, immune response, and organic substance metabolic process. The identified TRIM genes were located in intracellular and contractile fiber part. The MF of these TRIM genes was related to transferase activity, transcription coregulator activity, and molecular adaptor activity. We next performed the Reactome pathway, and seven significant pathways were identified, relying on the ConsensusPathDB database. As the results displayed in Figure 1F, the identified TRIM genes were associated with the immune system, interferon signaling, cytokine signaling, and adaptive immune system.

We explored the correlation among these common TRIM genes in TCGA and ICGC cohorts. Nine TRIMs, including TRIM16, TRIM45, and TRIM65 were found to be co-expressed in the TCGA cohorts (Figure 1G). In the ICGC cohort, TRIM47 was found to be correlated to TRIM65, TRIM17 correlated to TRIM50, and TRIM16 co-expressed to TRIM16L (Figure 1H). Based on the STRING database, we also assessed the interaction of these TRIM genes (Figure 1I). The results showed that 15 TRIM genes, including TRIM16L, TRIM37, and TRIM46, could a complex interaction networks.

Additionally, to assess our identified TRIM genes in HBV-associated HCC in TCGA and ICGC cohorts, the expression of the identified TRIMs was estimated in the GSE77509 and GSE94660 cohort, in which the gene expression was acquired from RNA-sequencing based on Illumina Hiseq platforms. The results were displayed in Figures 1J,K, and except TRIM54, TRIM62, and TRIM7, other TRIM genes were found to upregulate in the GSE77509 cohort. In the GSE94660 cohort, the increased expression levels of all identified 17 TRIM genes were verified in HBV-associated HCC (*p* < 0.05). The expressions of identified TRIMs were also measured using the GSE55092 and GSE121248 cohorts, in which the gene expression information was acquired by Affymetrix Human Genome U133 Plus 2.0 Array. As the results are shown in Figure 1L and M, TRIM16L was not identified in these two cohorts. Expect TRIM17, TRIM46, TRIM50, TRIM62, TRIM65, and TRIM7, the other 10 TRIMs, were found to upregulate in HBV-positive HCC in the GSE55092 cohort (*p* < 0.05). Besides these, a total of 9 TRIMs, excluding TRIM17, TRIM46, TRIM65, TRIM47, TRIM50, TRIM54, and TRIM60, were discovered to be increased in HBV-positive HCC in the GSE121248 cohort (*p* < 0.05).

### The Association of Identified TRIM Genes With Other E3 Ligase Genes in HBV-Related Hepatocellular Carcinoma

**FIGURE 2:**
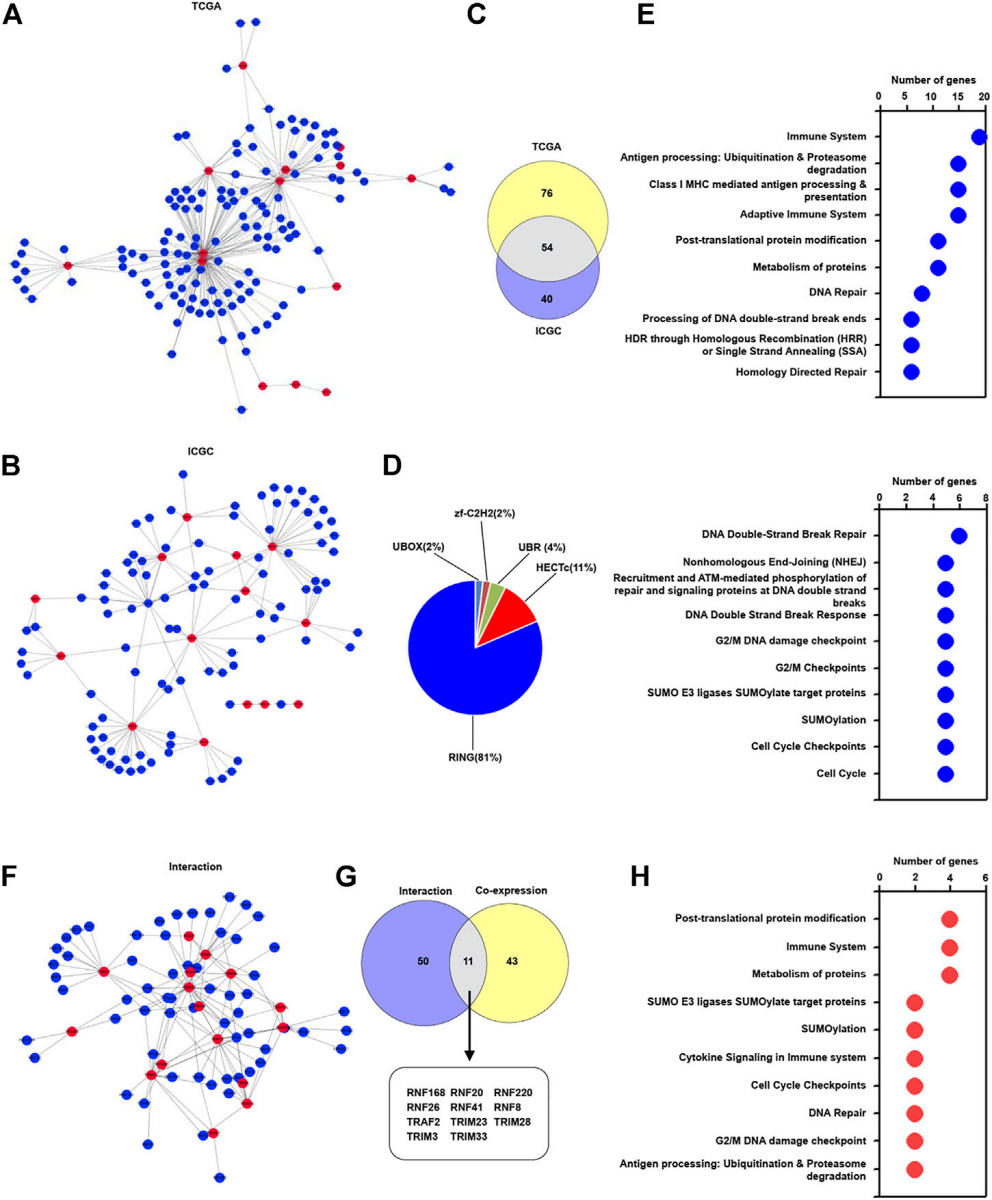
The correlation of TRIM genes with other E3 ligases in HBV-positive HCC. **(A)** The co-expression pattern of 17 TRIMs with other E3 ligases in HBV-associated HCC in TCGA cohort. **(B)** The co-expression pattern of 17 TRIM genes with other E3 ligases in HBV-associated HCC in TCGA and ICGC cohort. **(C)** The common co-expressed E3 ligases without 17 identified TRIMs in HBV-positive HCC in the ICGC cohort. **(D)** The proportion of the E3 ligases with different domains. **(E)** The terms of Reactome pathway analysis related to the common E3 ligases. **(F)** The interaction of 17 TRIMs with identified other E3 ligases. **(G)** The common genes between the co-expressed and interacted E3 ligases. **(H)** Reactome pathway analysis related to the common E3 ligases.

TRIMs belong to the E3 ligase family (Crawford et al., 2018), and different E3 ligases may regulate special biological processes in a collaborative manner. We interestingly explored the co-expression pattern of these identified TRIM genes with other E3 ligase genes to modulate special biological processes in HBV-associated HCC tissues. As the results are presented in Figure 2A, 130 E3 ligase genes were found to form a complex co-expression network with identified TRIM genes in TCGA cohorts. A total of 94 E3 ligase genes were discovered to interact with these identified TRIM genes in ICGC cohorts (Figure 2B). Additionally, 54 common E3 ligases were also found in both two cohorts (Figure 2C). Especially, among these common E3 ligases, most of the E3 ligase genes had RING domain, and other E3 ligase genes had HECTc, UBR, UBOX, and zf-C2H2 domains (Figure 2D). The pathways related to these common E3 ligase genes were also investigated. As the results displayed in Figure 2E and Supplementary Table S2, these E3 ligase genes were associated with the immune system, adaptive immune system, Antigen processing: Ubiquitination and Proteasome degradation, SUMOylation, Post-translational protein modification, and cell cycle. We also assessed whether these identified common E3 ligase genes could interact with these 17 TRIM genes. Based on the STRING database, 61 proteins were found to interact with these TRIM genes (Figure 2F). Furthermore, among these proteins which can interact with 17 TRIM genes, 11 common E3 ligase genes, including RNF168, RNF20, and RNF220, could co-expressed with identified TRIM genes (Figure 2G). Besides, these common E3 ligase genes were associated with the immune system, cytokine signaling in immune system and SUMOylation (Figure 2H and Supplementary Table S3).

### The Association of Identified TRIM Genes With Innate Immune Genes in HBV-Associated Hepatocellular Carcinoma

**FIGURE 3:**
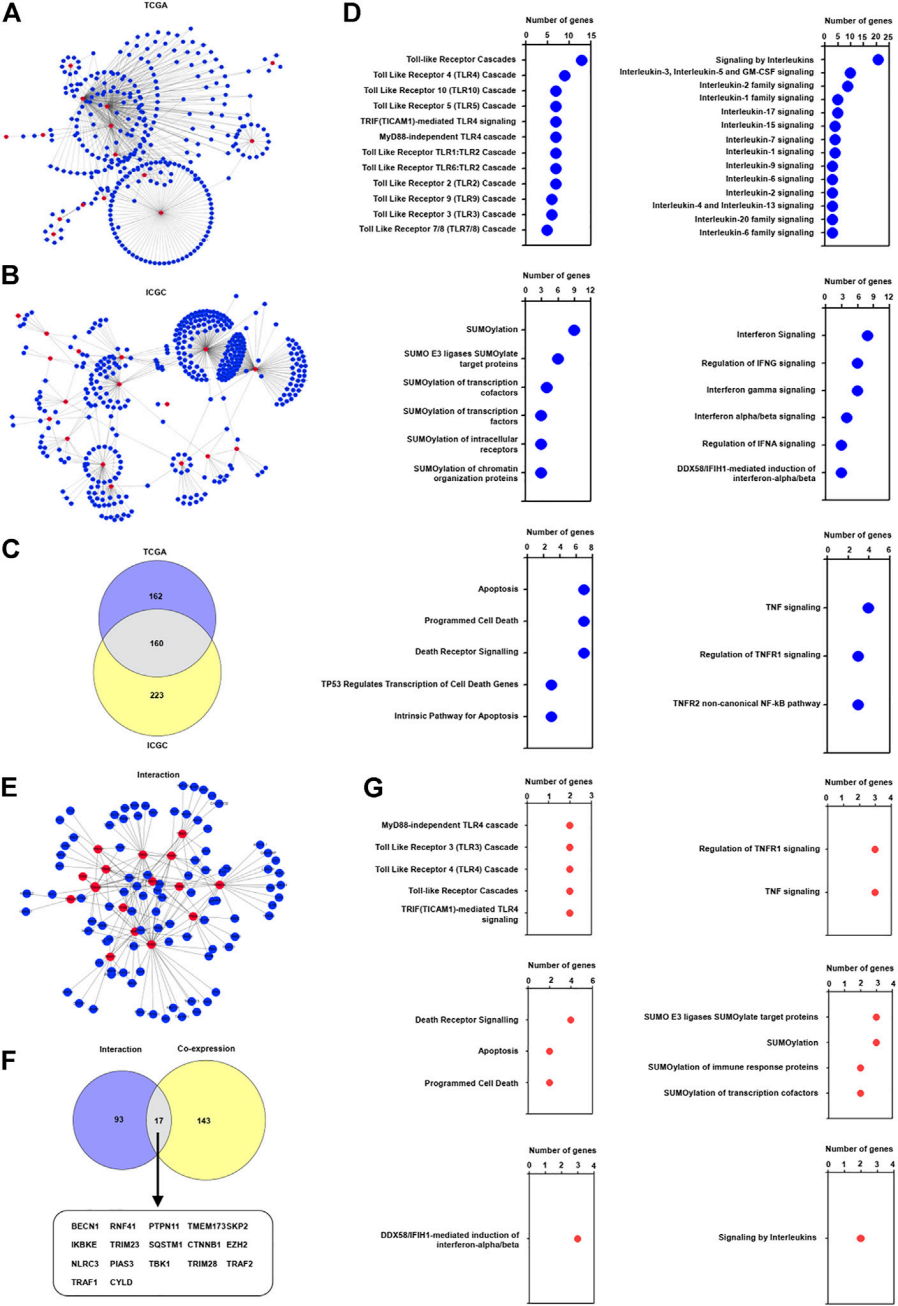
The correlation of TRIMs with innate immune genes in HBV-positive HCC. **(A)** The co-expression pattern of innate immune genes with 17 TRIMs in HBV-positive HCC in TCGA cohort. **(B)** The co-expression pattern of innate immune genes with 17 TRIMs in HBV-positive HCC in ICGC cohort. **(C)** The common co-expressed innate immune genes with identified TRIMs in HBV-associated HCC in both TCGA and ICGC cohorts. **(D)** The terms of Reactome pathway analysis related to the common innate immune genes. **(E)** The interaction of 17 TRIMs with identified innate immune genes. **(F)** The common genes between the co-expressed and interacted innate immune genes. **(G)** The terms of Reactome pathway analysis related to the common innate immune genes.

Current investigations indicated that TRIM proteins have a complicated effect on the innate immune response (Jin and Zhu, 2021), we are interested in investigating whether these identified TRIM genes participated in the innate immune response in HBV-associated HCC. Particularly, the correlation of the innate immune genes, which were identified from the InnateDB database (Breuer et al., 2013), with the identified 17 TRIM genes were assessed. As shown in Figure 3A, 322 innate immune genes were capable of constructing a complex co-expression network with the TRIMs in the TCGA cohort. A total of 383 innate immune genes were capable of co-expressing with these identified TRIM genes in the ICGC cohort (Figure 3B). Between these two cohorts, 160 common innate immune genes were discovered (Figure 3C). Furthermore, these common innate immune genes were related to interleukin signaling, interferon signaling, apoptosis, SUMOylation, and Toll-like receptor (TLR) signaling (Figure 3D and Supplementary Table S4). Based on the STRING database, 110 innate immune genes were found to interact with TRIM genes (Figure 3E). Furthermore, 17 common innate immune genes were discovered to co-express and interact with identified TRIM genes (Figure 3F). Besides, these common innate immune genes are associated with TNF/TNFR1 signaling, TLR signaling, SUMOylation, and death receptor signaling (Figure 3G).

### The Association of Identified TRIM Genes With Infiltrating Immune Cells, as Well as Immune Checkpoint Genes, in HBV-Associated Hepatocellular Carcinoma

**FIGURE 4:**
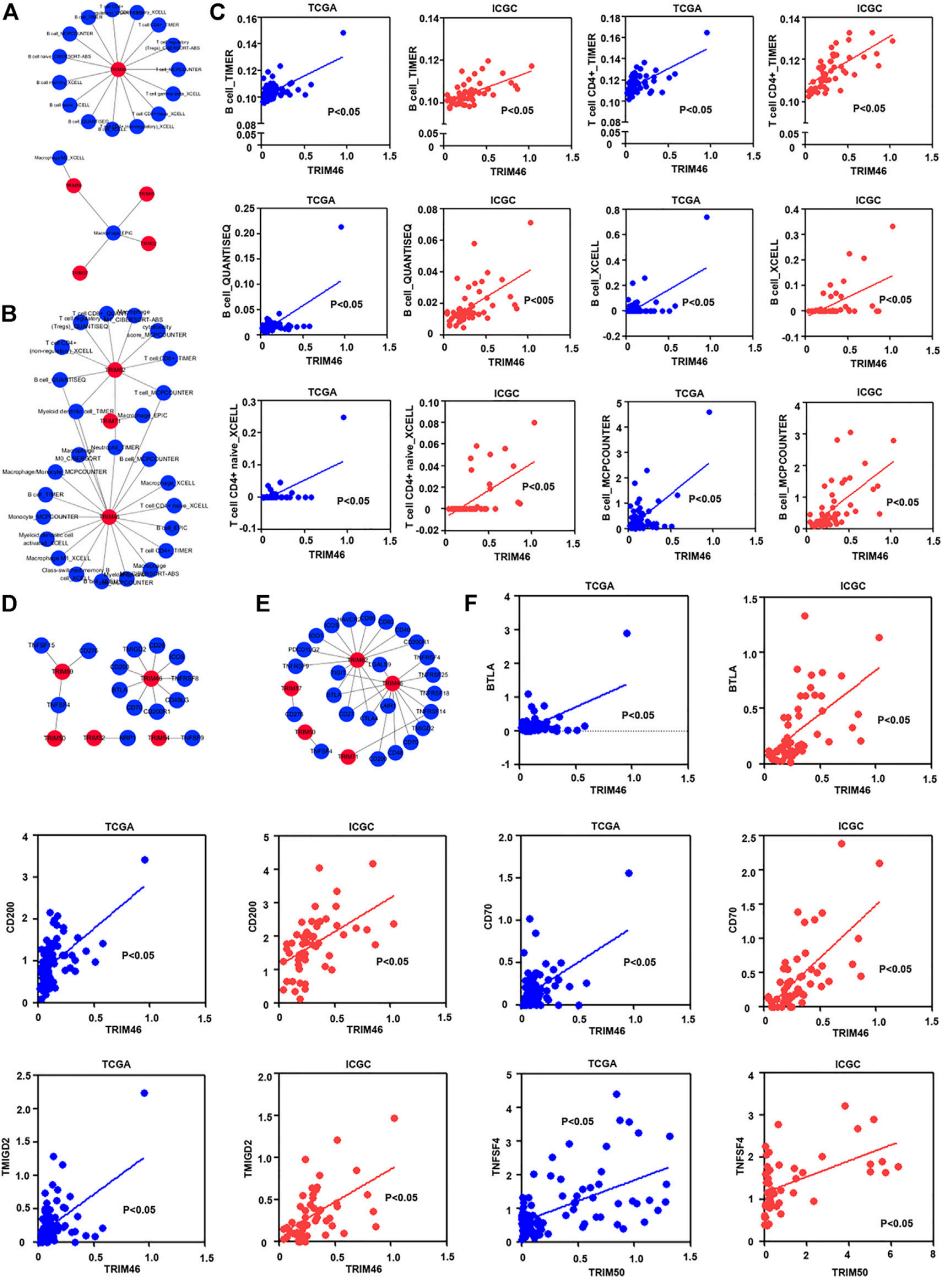
The correlation of TRIM genes with immune cells and immune checkpoint genes in HBV-positive HCC. **(A)**. The co-expression pattern of TRIMs with immune cells in HBV-positive HCC in TCGA cohort. **(B)** The co-expression pattern of TRIMs with immune cells in HBV-related HCC in ICGC cohort. **(C)** The common co-expression pattern of TRIM genes with immune cells in HBV-positive HCC in both two cohorts. **(D)** The co-expression pattern of TRIMs with immune checkpoint genes in HBV-associated HCC in TCGA cohort. **(E)** The co-expression pattern of TRIM genes with immune checkpoint genes in HBV-positive HCC in the ICGC cohort. **(F)** The common co-expression pattern of TRIM genes with immune checkpoint genes in HBV-associated HCC in TCGA and ICGC cohorts.

Current investigation has demonstrated that tumor immune infiltrating cells had a vital effect on tumor immune regulation (Mao et al., 2021; Xie et al., 2021). We also assess the correlations of identified TRIM genes with the infiltration of different immune cells. Depending on the TIMER2.0 database, the proportion of infiltrated immune cells in HBV-associated HCC was calculated. As the results displayed in Figure 4A, TRIM46 was relevant to the infiltration of T cells and associated subtypes. TRIM32, TRIM37, TRIM45, and TRIM59 were linked to macrophages in the TCGA cohort. Besides, in the ICGC cohort, TRIM71 was related to macrophages. TRIM46 and TRIM62 were related to T cells and B cells (Figure 4B). Between these two cohorts. TRIM46 was relevant to the infiltration of CD4+ T cells and B cells in HBV-associated HCC (Figure 4C).

It is well-known that immune checkpoints play vital roles in modulating the function of different immune cells (Li et al., 2021; Zheng et al., 2021). We also measured the correlations of TRIM genes with immune checkpoint genes. As the results are shown in Figure 4D, TRIM32, TRIM46, TRIM54, TRIM50, and TRIM59 could co-express with different immune checkpoint genes in the TCGA cohort. TRIM17, TRIM46, TRIM50, TRIM62, and TRIM71, were also discovered to correlate with different immune checkpoint genes in the ICGC cohort (Figure 4E). Between these two cohorts, TRIM46 was positively associated with CD70, BTLA, CD200, and TMIGD2. TRIM50 was positively related to TNFSF4 (Figure 4F).

### The Mutation Characteristics of TRIM Genes in HBV-Associated Hepatocellular Carcinoma

**FIGURE 5:**
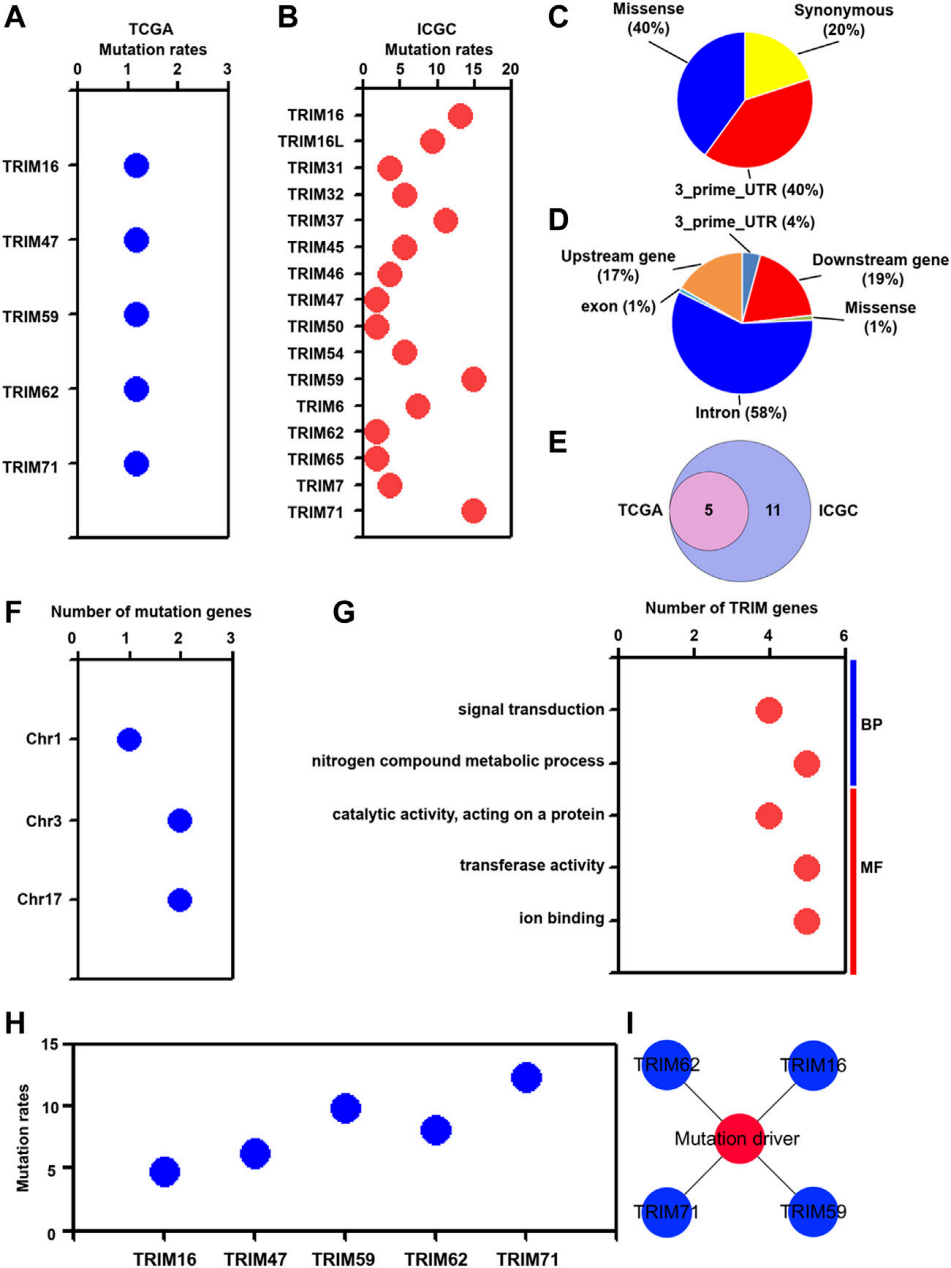
The mutation characteristics of TRIMs in HBV-positive HCC. **(A)** The mutation proportion of TRIMs in the TCGA cohort. **(B)** The mutation proportion of TRIMs in the ICGC cohort. **(C)** The mutation types and their mutation proportions in the TCGA cohort. **(D)** The mutation types and their mutation proportions in the ICGC cohort. **(E)** The number of commonly mutated TRIM genes in both TCGA and ICGC cohorts. **(F)** The colocation of TRIM genes in different chromosomes. **(G)** The GO terms of common mutated TRIMs. **(H)** The mutation proportion of common TRIMs in the LICA-CN cohort. **(I)** The mutation drivers are related to identified TRIMs.

It has been widely noticed that genetic mutations were closely related to oncogenesis and played a very critical role in hepatocarcinogenesis (An et al., 2018; Muller et al., 2020). We assessed the correlation of TRIM mutations with HBV-associated HCC. As presented in Figure 5A, TRIM16, TRIM47, TRIM59, TRIM62, and TRIM71 were found to be mutated in HBV-positive HCC in the TCGA cohort. In the ICGC cohort, 16 TRIM genes, including TRIM16, TRIM16L, and TRIM31, were found to have mutations in the ICGC cohort (Figure 5B). In the TCGA cohort, 3 mutation types, including synonymous, 3_prime_UTR, and missense mutations were found (Figure 5C). In the ICGC cohort, 6 mutation types, including 3_prime_UTR, upstream, downstream, missense, exon, and intron mutations were discovered (Figure 5D). Besides, 5 common TRIM genes had the mutations in these two cohorts (Figure 5E). Furthermore, these common TRIM genes were located in chr1, chr3, and chr17 (Figure 5F). Based on GO analysis, we found that the commonly mutated TRIM genes were related to signal transduction and nitrogen compound metabolic process in BP, and were associated with catalytic activity, transferase activity, and ion binding in MF (Figure 5G). To validate the common TRIM gene mutation in HBV-positive HCC, the LICA-CN cohort was utilized. As the result displayed in Figure 5H, the mutation data of these commonly mutated TRIM genes was also found in the LICA-CN cohort. Furthermore, based on the OncoVar online database, TRIM16, TRIM59, TRIM62, and TRIM71 genes were shown to act as tumor mutation drivers (Figure 5I).

### The Correlation of Identified TRIM Genes With the Clinicopathological Factors in HBV-Positive Hepatocellular Carcinoma

**FIGURE 6:**
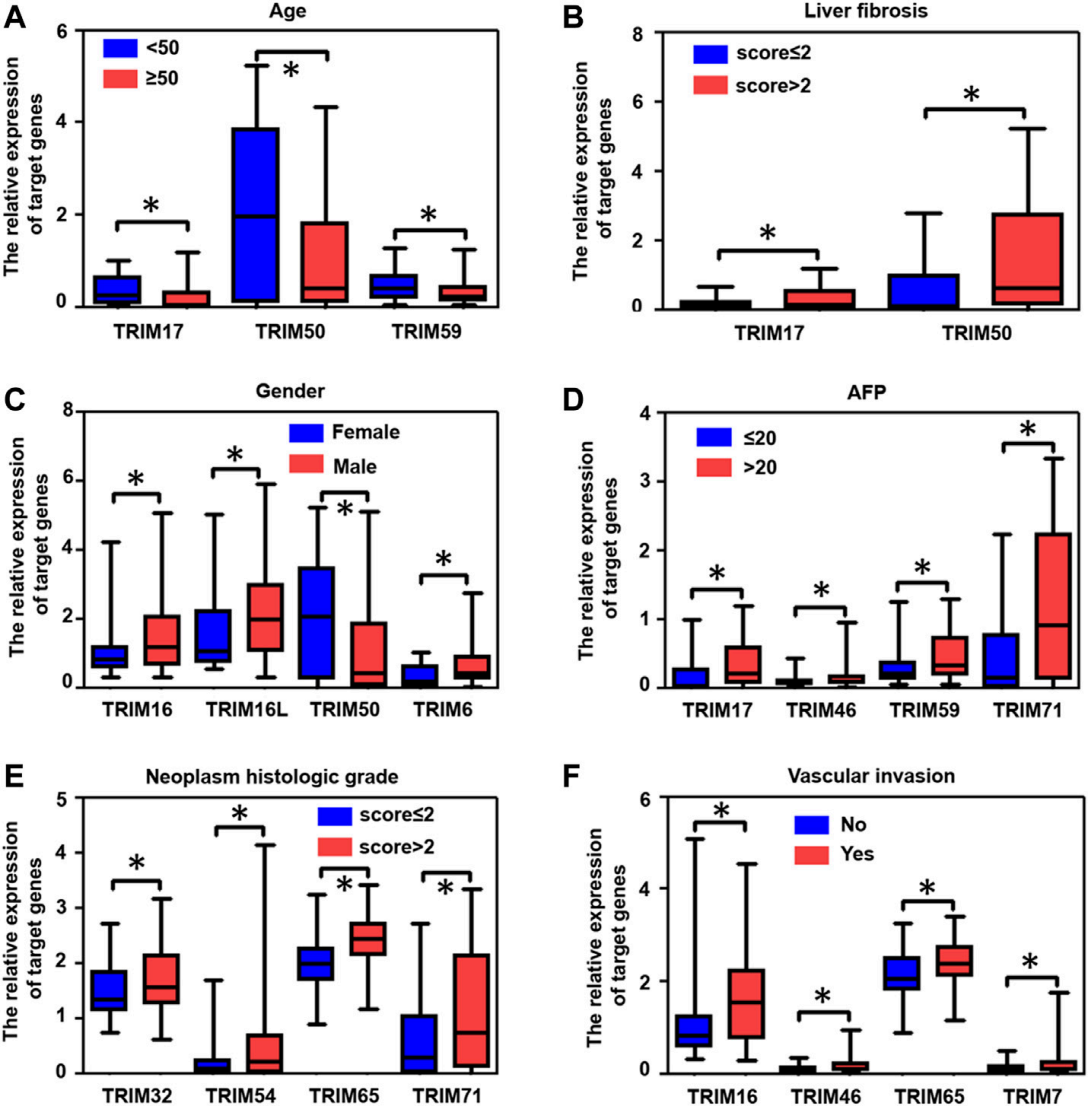
The association of identified TRIMs with different clinical factors in patients with HBV-positive HCC. **(A)** The correlation of TRIMs with ages in HBV-positive HCC patients. **(B)** The correlation of TRIMs with liver fibrosis in HBV- positive HCC patients. **(C)** The correlation of TRIMs with gender in HBV-positive HCC patients. **(D)** The correlation of TRIMs with AFP levels in patients with HBV-positive HCC. **(E)** The correlation of TRIMs with different neoplasm histologic grades in HBV-positive HCC patients. **(F)** The correlation of TRIMs with or without vascular invasion in patients with HBV-positive HCC.

Next, depending on the HBV-positive HCC in the TCGA cohort, we further evaluated the correlation of identified TRIM genes with multiple clinical factors, including Age, fibrosis, gender, AFP levels, neoplasm histologic grade, and vascular invasion. The results were shown in Figure 6A, in HBV-positive HCC patients with age ≥ 50, the expression levels of TRIM17, TRIM50, and TRIM59 were decreased, compared to these HBV-associated HCC patients with age < 50. Compared to the patients with fibrosis scores ≤ 2, TRIM17 and TRIM50 expressions were increased in patients with fibrosis scores > 2 (Figure 6B). In comparison with female HBV-associated HCC patients, the expression levels of TRIM16, TRIM16L, and TRIM6 were upregulated in male HBV-associated HCC patients (Figure 6C). Nevertheless, the expression levels of TRIM50 were declined in male HBV-related HCC patients. In comparison with HBV-related HCC with AFP levels ≤ 20, the expression levels of TRIM17, TRIM46, TRIM59, and TRIM71 were upregulated in patients with AFP > 20 (Figure 6D). Compared with patients with neoplasm histologic grades ≤ 2, the expression levels of TRIM32, TRIM54, TRIM65, and TRIM71 were upregulated in patients with neoplasm histologic grades > 2 (Figure 6E). Additionally, in HBV-positive HCC patients with vascular invasion, compared to patients without vascular invasion, the expression levels of TRIM16, TRIM46, TRIM65, and TRIM7 were upregulated (Figure 6F).

### The Correlation of Identified TRIMs With the OS and DFS of HBV-Associated Hepatocellular Carcinoma Patients

**FIGURE 7:**
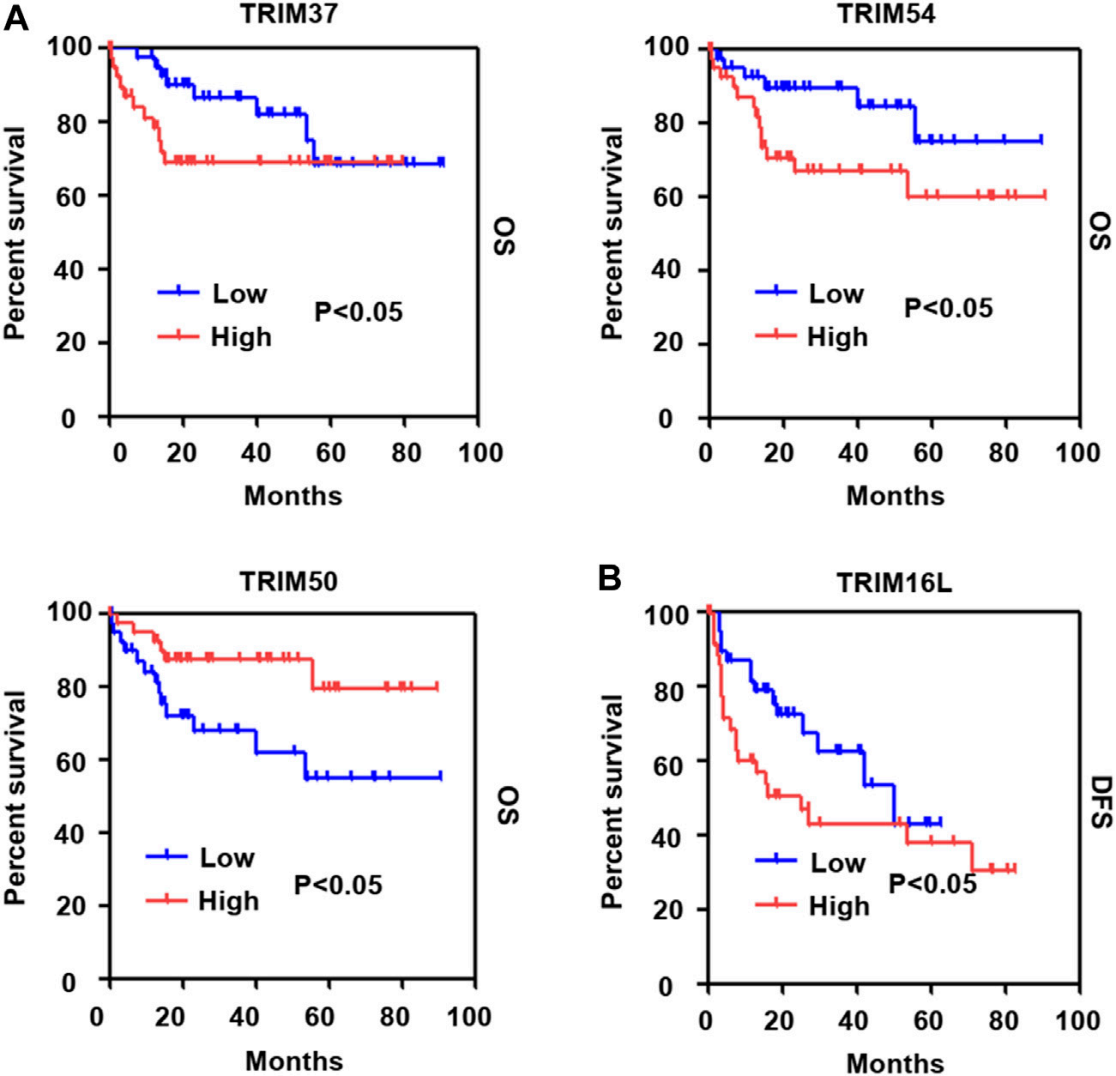
The association of TRIM genes with the survival in HBV-associated HCC based on the Kaplan-Meier analysis with the Genhan-Breslow-Wilcoxon test. **(A)** The relationships of TRIM37, TRIM50, and TRIM54 with OS of HBV-positive HCC patients. **(B)** The relationships of TRIM16L with DFS in HBV-associated HCC.

Next, dependent on the multivariate analysis, we measured the correlation of identified TRIM genes with OS and DFS of HBV-positive HCC in the TCGA cohort. As the results displayed in Table 1, TRIM50 was linked to the OS of HBV-associated HCC, TRIM16L, TIRM17, and TRIM6 were linked to the poor DFS of HBV-related HCC (Table 2). We also measured the relationship of these TRIMs with the survival of patients with HBV-related HCC, using the Genhan-Breslow-Wilcoxon test in Kaplan-Meier analysis. As displayed in Figure 7A. TRIM37 and TRIM54 were linked to the poor OS of patients with HBV-associated HCC. However, TRIM50 was found to be relevant to the beneficial OS of HBV-positive HCC patients. Besides, TRIM16L was linked to the poor DFS of patients with the virus related HCC (Figure 7B).

**TABLE 1:** The correlation of TRIM genes with the OS of HBV-positive HCC patients dependent on multivariate analysis in TCGA cohort.

Covariates	*p* value	Hazard radio	95.0% CI	
			up	down
TRIM16	0.821	1.201	0.246	5.863
TRIM16L	0.196	2.535	0.619	10.388
TRIM17	0.423	0.577	0.150	2.219
TRIM31	0.311	1.688	0.613	4.650
TRIM32	0.709	1.262	0.372	4.283
TRIM37	0.144	3.475	0.653	18.486
TRIM45	0.408	0.521	0.111	2.439
TRIM46	0.357	1.906	0.483	7.520
TRIM47	0.784	0.827	0.214	3.205
TRIM50	0.025	0.211	0.054	0.822
TRIM54	0.299	2.175	0.502	9.419
TRIM59	0.294	1.872	0.580	6.039
TRIM6	0.906	1.077	0.314	3.698
TRIM62	0.589	0.716	0.213	2.404
TRIM65	0.225	2.228	0.611	8.118
TRIM7	0.491	0.598	0.138	2.587
TRIM71	0.242	0.430	0.105	1.769

**TABLE 2:** The correlation of TRIM genes with the DFS of HBV-positive HCC patients dependent on multivariate analysis in TCGA cohort.

Covariates	*p* value	Hazard radio	95.0% CI	
			up	down
TRIM16	0.117	0.430	0.150	1.236
TRIM16L	0.010	4.291	1.421	12.961
TRIM17	0.006	0.258	0.097	0.684
TRIM31	0.460	0.734	0.323	1.668
TRIM32	0.313	1.614	0.637	4.090
TRIM37	0.304	0.556	0.182	1.703
TRIM45	0.061	2.560	0.958	6.843
TRIM46	0.658	0.811	0.321	2.048
TRIM47	0.773	0.856	0.299	2.454
TRIM50	0.506	0.690	0.231	2.059
TRIM54	0.260	0.555	0.200	1.544
TRIM59	0.117	2.300	0.811	6.519
TRIM6	0.018	2.791	1.193	6.533
TRIM62	0.101	0.467	0.188	1.160
TRIM65	0.054	3.153	0.979	10.150
TRIM7	0.632	0.760	0.248	2.330
TRIM71	0.826	0.899	0.346	2.331

## Discussion

HBV has been demonstrated to as a common etiological factor for hepatocarcinogenesis worldwide (Amaddeo et al., 2015; Arslan et al., 2021). Emerging studies have been indicated that the TRIM family not only contributed to the progression of HCC but also participated in the modulation of HBV infection (Fan et al., 2019; Liu et al., 2020; Mu et al., 2020), while to date, the relationship of TRIM with HBV-associated hepatocarcinogenesis is not well investigated. Here, we acquired the information with regard to the mRNA expression and somatic mutations of TRIM genes in HBV-positive HCC in both TCGA and ICGC cohorts. Dependent on these two cohorts and integrated bioinformatics analysis, 17 TRIM genes were discovered to be upregulated in HBV-associated HCC, and these TRIM genes were found to be correlated with other E3 ligase genes, several innate immune genes, and distinct immune cells. In addition, the mutation of some TRIM genes had been discovered in HBV-related HCC tissues. Furthermore, many TRIM genes were associated with different clinical factors, as well as the poor survival of HBV-associated HCC patients.

At present, based on the RNA sequencing data required from Illumina Hiseq platforms in the TCGA and ICGC database, 17 common differentially expressed TRIMs were identified, and all of these TRIMs were upregulated in HBV-related HCC. Besides these, 14 common TRIM genes were found to upregulate in GSE77509, and the expression of all common TRIMs was verified in the GSE94660, in which the gene expression levels of identified TRIMs were also measured by the Illumina platform-dependent RNA sequencing. However, only 10 TRIMs and 9 TRIMs were found to upregulate in the GSE55092 and GSE121248 cohorts, in which the expression levels of identified TRIMs were obtained by Affymetrix microarray platforms. The researches from different groups have been demonstrated that RNA-sequencing has better sensitivity than microarray to detect differentially expressed genes (Xu et al., 2013; Vahlensieck et al., 2021), and this may explain the upregulated TRIMs, including TRIM17, TRIM46, and TRIM50, could not be identified by not all microarray data but could be discovered by RNA sequencing. In the future, the gene and protein levels of these identified TRIMs presented in the study should be further confirmed by different methods to assess the exact relationship of TRIMs with HBV-positive HCC and examine the biological function of TRIMs on the development of HBV-associated HCC. Dependent on the GO analysis, the identified TRIMs were displayed to be linked to a variety of BP, CC, and MF. For example, the identified TRIMs were linked to the cellular metabolic process, immune response, and nitrogen compound metabolic process. In addition, the identified TRIM genes were situated in intracellular and contractile fiber part. Furthermore, these TRIM genes were related to transferase activity, transcription coregulator activity, and molecular adaptor activity. Besides these, the enrichment analysis from the KEGG pathway indicated that these identified TRIM genes were not only related to the immune system, and interferon and cytokine signaling but also relevant to the adaptive immune system. Moreover, many of these identified TRIMs could interplay with each other and form a complex interaction network. Besides, some TRIM genes were capable of co-expressing with other genes in HBV-associated HCC. These findings implied that the identified TRIMs may exert their functions in a co-expression manner and further interact with each other to modulate the immune response to facilitate the progress of HBV-associated HCC.

As essential components of E3 ligases (Venuto and Merla, 2019; Mohammadi et al., 2021), TRIMs may collaborate with other E3 ligases to regulate the biological functions of HBV-associated HCC. We explored whether the 17 common E3 ligases could co-express with other E3 ligase genes in the TCGA and ICGC cohorts. The results indicated that these TRIMs were capable of co-expressing with many other E3 ligases, and these E3 ligases were associated with the immune system, adaptive immune system, SUMOylation, and post-translational protein modification. Furthermore, based on the STRING database, we found that these TRIM genes could interact with other E3 ligases. These results suggested that these TRIM genes could regulate immune response and other biological processes through cooperating with other E3 ligases in HBV-positive HCC.

TRIM genes are essential for innate immune response (Jin and Zhu, 2021), and we assessed the relationship of identified TRIM genes with innate immune genes in HBV-positive HCC. The results indicated that these TRIMs were able to co-express with a variety of innate immune genes, and these identified innate immune genes were associated with interleukin signaling, interferon signaling, apoptosis, SUMOylation, and TLR signaling. Moreover, these TRIMs also can interact with many innate immune genes to contribute to the regulation of several pathways, including interleukin signaling, and TLR signaling. Together, these results indicated that the identified TRIMs may interact with other innate immune genes with various biological functions to modulate the innate immune response to participate in HBV-associated hepatocarcinogenesis.

It is worth noting that the development and metastasis of cancer are related to tumor-infiltrating immune cells (Xie et al., 2021). Furthermore, the dysfunction of the adaptive immune responses mediated by various immune cells had critical roles in hepatocarcinogenesis during HBV infection (Cho and Cheong, 2021). We investigated the association of identified TRIMs with the infiltration of different immune cells, and as the results were presented in Figure 4, TRIM46 was significantly related to the infiltration of CD4+ T cells and B cells. Because the immune checkpoint molecules have a very vital effect on immune cells activation, we also assessed the correlation of immune stimulate molecules with identified TRIMs, our results indicated that TRIM46 was associated with CD70, BTLA, and TRIM50 correlated to TNFSF4. These results implied that the identified TRIMs may modulate the activation of T cells and B cells through different immunostimulatory factors in HBV-associated HCC. Taken together, our immune-related results indicated that these TRIMs might participate in the regulation of the adaptive immune response mediated by T cells and B cells to modulate the progress of HBV-positive HCC.

It has been reported that gene mutation is essential for the development of HBV-associated HCC (Zhan et al., 2017). Among these identified TRIMs, the mutation of 5 common TRIM genes was discovered in HBV-positive HCC. In addition, the results of GO analysis suggested that these mutated TRIMs were related to signal transduction and nitrogen compound metabolic process. Furthermore, among these mutated TRIM genes, 4 TRIMs, including TRIM16, TRIM59, TRIM61, and TRIM71 were identified as mutation driver genes. These results implied that the mutated TRIMs may have a vital role in HBV-associated hepatocarcinogenesis.

It is widely noted that several genes are capable of being applied as promising diagnostic and prognostic biomarkers for HCC (Jiang W. et al., 2021; Zhou et al., 2021). The association of identified TRIMs with clinicopathological factors and the prognosis of HBV-associated HCC was investigated. Based on TCGA cohorts, our results suggested that many TRIMs were related to distinct clinical parameters, including age, fibrosis, gender, AFP levels, neoplasm histologic grades, and vascular invasion. The multivariate analysis shows that TRIM50 was related to the OS of HBV-positive HCC. TRIM16L, TRIM17, and TRIM6 were associated with the DFS of the disease. Depending on Kaplan-Meier analysis with Genhan-Breslow-Wilcoxon test, TRIM37, TRIM50, and TRIM54 were found to be relevant to the OS of HBV-positive HCC. Additionally, TRIM16L was discovered to be linked to the poor DFS of the disease. These results suggest that these TRIMs could be utilized as biological biomarkers of the poor prognosis of HBV-positive HCC patients. Moreover, the integration of the expression data of identified TRIMs with appropriate approach may be a favorable strategy for managing the disease to abate the mortality of HBV-positive HCC patients.

## Conclusion

Taken together, dependent on integrative analysis, we identified 17 TRIM genes that were involved in the progress of HBV-positive HCC in the TCGA and ICGC cohorts. Our current results indicated that all of these TIRMs were increased, and can form a complex interaction and co-expression networks in HCC with HBV infection. Besides, some of the identified TRIM genes correlated to other E3 ligase genes, innate immune genes, and immune cell infiltration. Furthermore, among these identified TRIMs, some were mutated in HBV-related HCC and identified as cancer driver genes. Moreover, many TRIM genes were linked to distinct clinical factors and associated with the poor progress of HBV-positive HCC. Furthermore, given the biological and clinical significance of identified TRIM genes, the identified TRIMs could act as novel molecular biomarkers or therapeutic targets for HBV-positive HCC. Here, the identified TRIMs in HBV-associated HCC were acquired from RNA-sequencing. However, not all of the identified TRIMs were verified in the HBV-related HCC cohorts detected by microarrays. The discrepancy in gene monitoring platforms with different sensitivity and accuracy may be associated with the inconsistency of the expression patterns of TRIM genes in different HBV-associated HCC cohorts. Nevertheless, more work by utilizing different methods is required to assess the exact reasons that caused the difference in gene expression in the identified TRIMs. Besides these, the predicted molecular functions and pathways related to the TRIM genes, including interleukin signaling, interferon signaling, apoptosis, SUMOylation, and TLR signaling, were generated from bioinformatics analysis. Relying on *in vitro* and *in vivo* experiments, the functional significance of these identified TRIM needs to be elucidated. Despite these limitations, our findings still lay a foundation for investigating the biological functions and related mechanisms modulated by the identified TRIMs in HBV-associated tumor.

## Data Availability

The datasets presented in this study can be found in online repositories. The names of the repository/repositories and accession number(s) can be found in the article/Supplementary Material.
